# Proton Magnetic Resonance Spectroscopy in 22q11 Deletion Syndrome

**DOI:** 10.1371/journal.pone.0021685

**Published:** 2011-06-30

**Authors:** Fabiana da Silva Alves, Erik Boot, Nicole Schmitz, Aart Nederveen, Jacob Vorstman, Christina Lavini, Petra Pouwels, Lieuwe de Haan, Don Linszen, Therese van Amelsvoort

**Affiliations:** 1 Department of Psychiatry, Academic Medical Centre Amsterdam, Amsterdam, The Netherlands; 2 Ipse de Bruggen, Centre for People with Intellectual Disability, Zwammerdam, The Netherlands; 3 Department of Radiology, Academic Medical Centre Amsterdam, Amsterdam, The Netherlands; 4 Department of Psychiatry, Rudolf Magnus Institute of Neuroscience, University Medical Centre Utrecht, Utrecht, The Netherlands; 5 Department of Physics and Medical Technology, VU University Medical Centre Amsterdam, Amsterdam, The Netherlands; 6 Arkin Mental Health Care, Amsterdam, The Netherlands; Rikagaku Kenkyūsho Brain Science Institute, Japan

## Abstract

**Objective:**

People with velo-cardio-facial syndrome or 22q11 deletion syndrome (22q11DS) have behavioral, cognitive and psychiatric problems. Approximately 30% of affected individuals develop schizophrenia-like psychosis. Glutamate dysfunction is thought to play a crucial role in schizophrenia. However, it is unknown if and how the glutamate system is altered in 22q11DS. People with 22q11DS are vulnerable for haploinsufficiency of PRODH, a gene that codes for an enzyme converting proline into glutamate. Therefore, it can be hypothesized that glutamatergic abnormalities may be present in 22q11DS.

**Method:**

We employed proton magnetic resonance spectroscopy (^1^H-MRS) to quantify glutamate and other neurometabolites in the dorsolateral prefrontal cortex (DLPFC) and hippocampus of 22 adults with 22q11DS (22q11DS SCZ+) and without (22q11DS SCZ−) schizophrenia and 23 age-matched healthy controls. Also, plasma proline levels were determined in the 22q11DS group.

**Results:**

We found significantly increased concentrations of glutamate and myo-inositol in the hippocampal region of 22q11DS SCZ+ compared to 22q11DS SCZ−. There were no significant differences in levels of plasma proline between 22q11DS SCZ+ and 22q11DS SCZ−. There was no relationship between plasma proline and cerebral glutamate in 22q11DS.

**Conclusion:**

This is the first *in vivo* ^1^H-MRS study in 22q11DS. Our results suggest vulnerability of the hippocampus in the psychopathology of 22q11DS SCZ+. Altered hippocampal glutamate and myo-inositol metabolism may partially explain the psychotic symptoms and cognitive impairments seen in this group of patients.

## Introduction

Velo-cadio-facial-syndrome or 22q11 deletion syndrome (22q11DS) is a genetic syndrome caused by a deletion on chromosome 22 which is accompanied by several somatic, behavioral, cognitive and psychiatric problems, and structural and functional brain abnormalities [1]. The estimated prevalence of 22q11DS in the general population is 1 in 5950 births [2]. Adults with 22q11DS face a 25 times higher risk of developing schizophrenia than the general population [3] and in people with schizophrenia an increased frequency of 22q11 deletions has been reported [4], [5]. Hence, a 22q11 deletion is among the highest risk factors for the development of schizophrenia.

People with 22q11DS are vulnerable to haploinsufficiency of approximately 30 genes located on the deleted region of chromosome 22q11, including the proline dehydrogenase gene (PRODH) [6]. This gene, which encodes for the PRODH enzyme also called proline oxidase (POX), is involved in converting proline to glutamate [7]. Dysfunction or genetic variations of the PRODH gene, and consequent hyperprolinemia, have been associated with susceptibility to schizophrenia and with learning disabilities [8]–[13]. In fact, proline has been shown to function as modulator of glutamate neurotransmission through NMDA receptors [14], [15] and dysregulation of the glutamatergic system has been widely implicated in schizophrenia.

The involvement of glutamate in schizophrenia is particularly related to NMDA receptor hypofunction. Evidence for the role of NMDA receptor hypofunction in schizophrenia comes from pharmacological studies of phencyclidine (PCP) and ketamine. These NMDA receptor antagonists have shown to produce schizophrenia-like behaviors in rodents [16]; to induce positive and negative symptoms in healthy humans [17]; and to aggravate psychotic symptoms in patients with schizophrenia [18]. Glutamate also plays a role in synaptic plasticity via NMDA receptors mediating higher cognitive functions such as learning and memory. NMDA receptor dysfunction has also been implicated in the cognitive deficits of schizophrenia [19]. In these people, agents that enhance NMDA receptor activity have shown to improve negative symptoms and to facilitate memory consolidation [20].

The brain areas associated with NMDA receptor hypofunction in schizophrenia include the prefrontal cortex and hippocampus [21]–[24]. The relationship between NMDA receptor hypofunction and glutamate release is not fully understood. NMDA hypofunction in schizophrenia could be related to insufficient or excessive glutamate release which may also differ between brain regions [25]. Increased glutamate exposure and its duration could explain the psychotoxic effects in schizophrenia.

Proton Magnetic Resonance Spectroscopy (^1^H-MRS) is a feasible method for *in vivo* quantification of glutamate concentration and other brain metabolites that, if altered, may reflect abnormal neuro-developmental features [26]. In schizophrenia an increasing number of ^1^H-MRS studies have been conducted. Although inconclusive, ^1^H-MRS findings also suggest abnormal glutamatergic neurotransmission [27]–[29].

To date, the glutamatergic system in 22q11DS has not been investigated. People with 22q11DS have an increased prevalence of schizophrenia and similar neuroanatomical abnormalities. Hence, in this study we employed ^1^H-MRS to measure glutamate in the dorsolateral prefrontal cortex and hippocampus in 22q11DS patients with (22q11DS SCZ+) and without schizophrenia (22q11DS SCZ−). We hypothesized altered glutamate concentrations in individuals with 22q11DS SCZ+ compared to healthy individuals and, in 22q11DS SCZ+ compared to 22q11DS SCZ−. Besides glutamate, we also analyzed other neurometabolites from ^1^H-MRS spectra including *N*-acetylaspartate, choline, myo-inositol and creatine which reflect the status of neuronal functioning and glial cells, possibly disturbed in 22q11DS.

Furthermore, we assessed levels of plasma proline and plasma glutamine in the 22q11DS group. Increased proline has been reported in 22q11DS patients [30]. In children with 22q11DS there was a relationship between increased plasma proline and decreased brain function [31]. High levels of proline in 22q11DS, consequence of POX deficiency, may be related to glutamate dysfunction particularly in 22q11DS SCZ+. Hence, we expected that plasma proline will be increased in 22q11DS SCZ+ and that it will correlate with glutamate concentrations in the brain.

## Materials and Methods

### Subjects

We included 22 adults with 22q11DS (mean ± SD) (22q11DS SCZ+ *n* = 12, age 29.25±8.24; 22q11DS SCZ− *n* = 10, age 28.50±8.47) and 23 healthy controls (HC, age 31.22±9.58).

Individuals with 22q11DS were recruited through the Dutch 22q11DS family association and through the departments of three Dutch Clinical Genetics centers. Healthy volunteers were recruited by local advertisement. The study was conducted at the Department of Psychiatry, Academic Medical Centre Amsterdam (AMC), The Netherlands and was approved by the Medical Ethics Testing Committee/AMC. All participants were capable of giving written informed consent and did so, after receiving full information on the study.

All individuals with 22q11DS were assessed by an experienced psychiatrist and a physician for people with an intellectual disabilities using available information from medical records and a semi-structured psychiatric interview. All diagnoses reported are DSM-IV diagnoses (American Psychiatric Association, 1994). The 22q11DS group was subdivided into 2 groups: those who were fulfilling DSM-IV criteria for schizophrenia (22q11DS SCZ+) all taking antipsychotic medication and having duration of illness >1 year (dose ranges and haloperidol equivalents [32] are displayed in Table 1) and those who did not have a past or current psychiatric history and had never taken antipsychotic or stimulant medication (22q11DS SCZ−).

**Table 1:** Medication and dosage taken by 22q11DS patients with schizophrenia.

Drugs	Dosis (mg/d)	Haloperidol equivalent (mg/d)^a^	N
Aripiprazole	5–15	1–7.5	3
Atomoxetine^b^	80		1
Clozapine	200–300	4–6	2
Methylphenidate^c^	36		1
Olanzapine	5	2.5	1
Quetiapine	50	0.5	2
Risperidone	3–4	5–6.7	2
Zuclopentixol	6	1.2	1

a Haloperidol equivalents derived from kane et al (2003).

b One patient took an antipsychotic and a selective norepinephrine inhibitor.

c One patient took an antipsychotic and a psychostimulant drug.

In addition, the Positive and Negative Symptom Scale (PANSS) [33] was used to assess positive, negative and general psychopathology in the 22q11DS SCZ+ group. The PANSS includes 30 items, subdivided in three categories: positive symptoms, negative symptoms and general psychopathology. A patient who rates “absent” (or 1) on all items would receive a total score of 30 and a subject who rates “extreme” (or 7) on all 30 items would receive a total score of 210. All patients underwent a formalized clinical interview of 35–40 minutes and the questions were in regard to the last two weeks.

For assessment of intelligence quotient (IQ) we used the shortened Dutch version of the Wechsler Adult Intelligence Scale (WAIS-III–NL) consisting of 5 subtests: vocabulary, comprehension, similarities (verbal IQ), block design, and object assembly (performance IQ) [34], [35].

All healthy volunteers were seen by a physician. They were included in the study after screening for psychiatric disorders and medical conditions affecting the brain. None of the participants had a history of substance or alcohol abuse. Urine drug screening (cocaine, tetrahydrocannabinol, opiates, amphetamines, benzodiazepines) was performed at study day and was negative in all subjects. Healthy participants were not using any medication at the time of testing.

### 1H-MR spectroscopy acquisition

**Figure 1:**
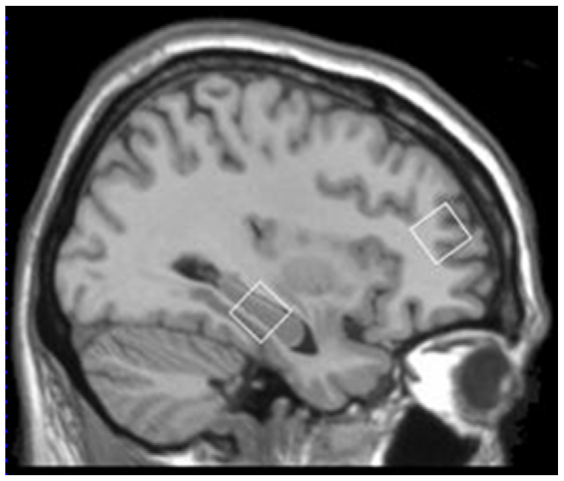
Sargittal T1-weighted magnetic resonance image of the brain showing voxel (2×2×2 cm) placement for proton magnetic resonance spectroscopy (1H-MRS) in the left dorsolateral prefrontal cortex and left hippocampus.

^1^H-MRS data acquisition took place at the Department of Radiology (Academic Medical Centre Amsterdam, The Netherlands) using a 3 Tesla Intera MRI system (Philips, Best, The Netherlands) equipped with a 6 channel sense head coil. For estimation of metabolite concentrations, two single 8 ml voxels of interest positioned in the left dorsolateral prefrontal cortex (DLPFC) (2×2×2 cm) and left hippocampus (2×2×2 cm) were obtained for each subject (Figure 1). More specifically, the hippocampal voxel included areas of the hippocampus, parahippocampal gyrus, fusiform gyrus and collateral sulcus. Iterative first order shimming was performed and water suppressed spectra was acquired using a point-resolved spatially localized spectroscopy sequence (PRESS, TE 36 ms, TR 2000 ms, 128 averages).

For anatomical localization transversal high-resolution structural T1-weighted volumetric images, with full head coverage, using 130 contiguous slices (1.2 mm thick, with 0.89×0.89 mm in-plane resolution) and a TR/TE of 9.8/4.5 milliseconds (flip angle 8″, FOV 224 cm) were obtained.

**Figure 2:**
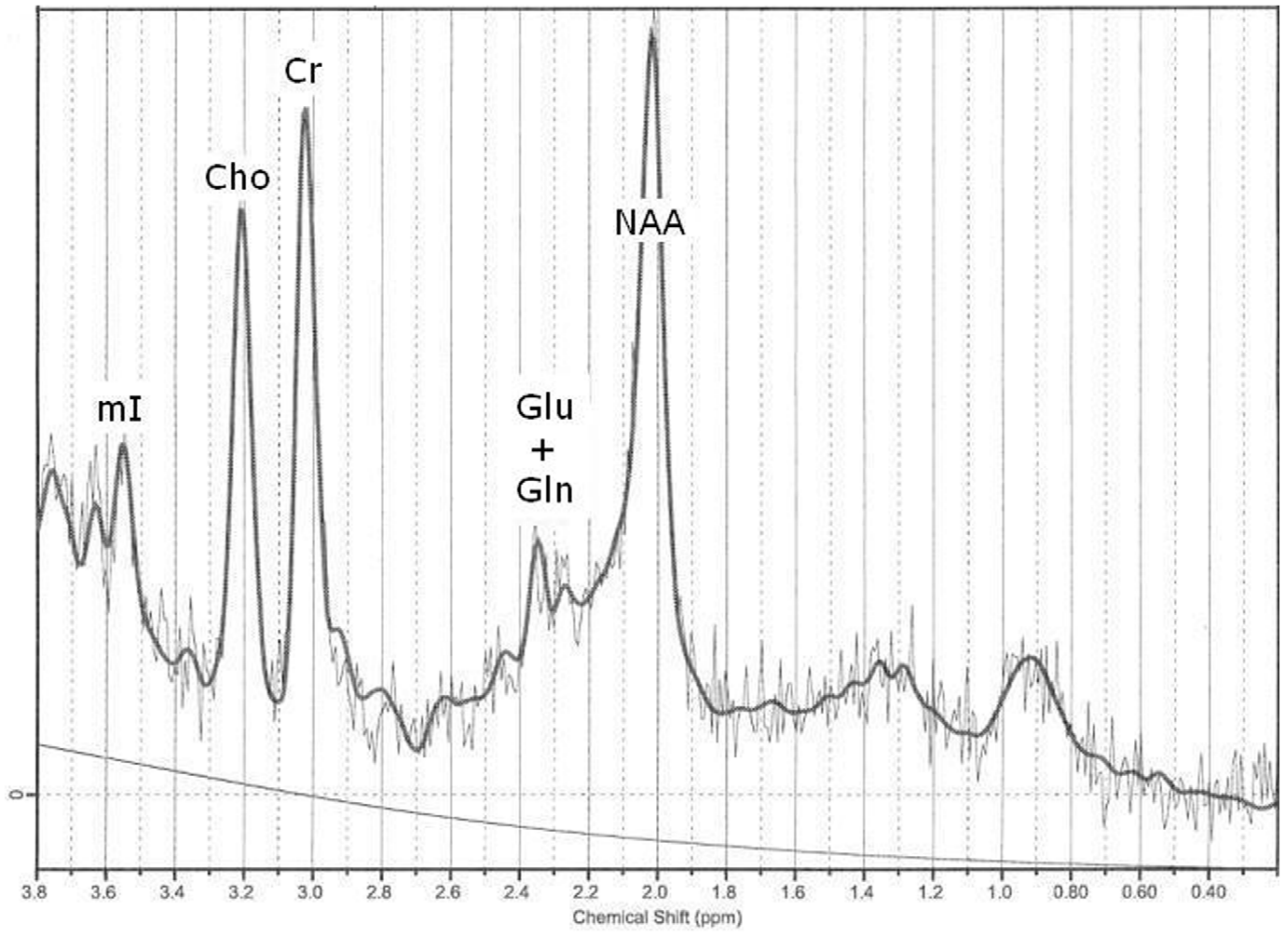
Sample of a 1H-MRS spectrum from hippocampus of a patient with 22q11DS as fit by LCModel.

^1^H-MRS spectra were analyzed using the Linear Combination of Model spectra (LCModel) commercial spectral-fitting package [36]. LCModel used a library of reference spectra in a basis set recorded specifically for the scanner and calibrated using the tissue water signal as an internal standard. The spectra were analyzed with a range of 3.8 ppm to 0.2 ppm (Figure 2). From the metabolites included in the LCModel basis set, we analyzed absolute levels of creatine *plus* phosphocreatine (Cr), glycerophosphocholine *plus* phosphocholine (choline), *myo-inositol*, *N*-acetylaspartate (NAA), NAA *plus N*-acetylaspartylglutamate (NAAG), glutamine and glutamate.

In addition, we analyzed the combination of glutamate *plus* glutamine (Glx). Glutamate and glutamine are closely related amino acids involved in intermediary metabolism, protein synthesis and neurotransmission. Metabolite concentrations are expressed in millimoles per liter.

Data were excluded from analysis if the voxel coordinates were not or incorrectly recorded. Spectral width (full width at half maximum, FWHM) was always lower than 0.1 p.p.m. and signal to noise ratio (SNR) greater than 11 as estimated by LCModel. Cramer-Rao minimum variance bounds (SD) was lower than 50% for glutamine and lower than 15% for the other metabolites.

### Plasma amino-acid analyses

Plasma proline and plasma glutamine concentrations of the 22q11DS group were assessed by automated ion exchange chromatography with post-column ninhydrin derivatization. Plasma amino-acid analyses were performed on a JEOL AminoTac (JEOL AminoTac JLC-500/V, Tokyo, Japan) following a morning blood draw.

### Statistical analyses

We used non-parametric Kruskal-Wallis H test to compare metabolite concentrations, age and IQ between the 3 groups (HC, 22q11DS SCZ+ and 22q11DS SCZ−) because the assumption of normal distribution was not met. Following, Post Hoc analyses were conducted with Mann-Whitney U tests. Correlation analyses were conducted with Spearman's rho test. Results are reported as significant when *P*≤0.05 (2-tailed). Statistical analyses were performed with SPSS, release 16.0.2 for Windows (SPSS Inc., Chicago, IL, USA. 2008).

## Results

### Demographics

Patients and healthy controls did not differ with regard to sex (HC 12m/11f, 22q11DS SCZ+ 8m/4f, 22q11DS SCZ− 4m/6f *P* = 0.45) and age (HC 31.22±9.51, 22q11DS SCZ+ age 29.25±8.24, 22q11DS SCZ− 28.50±8.47; *P* = 0.89).

Patients had a lower total IQ than healthy controls (HC 111.88±14.82, 22q11DS SCZ+ 69.67±13.82, 22q11DS SCZ− 81.86±7.01; *P*<0.001). Also verbal IQ (HC 112.88±15.96, 22q11DS SCZ+ 75.00±11.24, 22q11DS SCZ− 85.86±9.33; *P* = 0.001) and performance IQ (HC 109.38±19.91, 22q11DS SCZ+ 67.89±16.60, 22q11DS SCZ− 79.43±10.53; *P* = 0.002) were significantly different between the groups. Post hoc analysis showed that HC compared to 22q11DS SCZ+ differed significantly for total IQ *P* = 0.001, verbal IQ *P* = 0.001 and performance IQ *P* = 0.001. HC compared to 22q11DS SCZ− differed significantly for total IQ *P* = 0.004, verbal IQ = *P* = 0.005 and performance IQ *P* = 0.01. 22q11DS SCZ+ compared to 22q11DS SCZ− differed significantly for total IQ *P* = 0.02 and verbal IQ *P* = 0.02 but not performance IQ *P* = 0.17.

For the 22q11DS SCZ+ group, the mean score on the general psychopathology PANSS subscale was 30.69±11.94, the negative subscale was 17.55±8.21 and the positive subscale was 10.69±3.81. The mean of total PANSS score was 58.95±21.85.

### Metabolites

Metabolite concentrations for the DLPFC and hippocampal region are displayed in Table 2. Kruskal-Wallis H test showed no significant group differences in any of the metabolite concentrations in the DLPFC. In the hippocampal region, significant group differences were found in concentrations of glutamate (*P* = 0.03) Glx (*P* = 0.03) and myo-inositol (*P* = 0.03). Post Hoc analysis indicated that these metabolite concentrations were significantly higher in 22q11DS SCZ+ compared to 22q11DS SCZ− patients (glutamate *P* = 0.02; Glx *P* = 0.03 and myo-inositol *P* = 0.01). Hippocampal Glx was higher in 22q11DS SCZ+ compared to HC (*P* = 0.02). In the DLPFC there was a significant positive correlation between glutamine concentration and antipsychotic dosage (n = 10 ρ = 0.64 *P* = 0.05) and a trend towards a positive correlation between Glx and antipsychotic dosage (n = 10 ρ = 0.59 *P* = 0.07). There were no significant correlations between hippocampal metabolites and antipsychotic dosage.

**Table 2:** Metabolites concentrations (mean/SD) in the DLPFC and hippocampal region in healthy controls and 22q11DS with and without psychosis.

DLPFC	*HC*	*SCZ−*	*SCZ+*	HIP	*HC*	*SCZ−*	*SCZ+*
*n = *	*23*	*7*	*11*	*n = *	*16*	*7*	*9*
Glu	6.44/1.35	6.35/1.02	6.39/1.32	Glu^a^	6.26/0.65	5.71/0.94	6.99/1.04
Gln	2.86/0.94	2.66/0.83	3.25/1.37	Gln	3.03/0.83	3.12/0.58	3.88/1.67
Glx	9.17/2.06	8.64/1.29	9.65/2.28	Glx^a^ ^b^	9.29/0.94	8.83/1.11	10.87/1.66
mI	3.51/0.54	3.35/0.50	3.46/0.83	mI^a^	3.87/0.63	3.47/0.40	4.43/0.76
NAA	6.07/0.79	5.38/0.63	5.89/0.82	NAA	5.03/0.57	4.63/0.85	5.25/1.18
NAA+NAAG	6.68/0.82	5.96/0.92	6.41/1.11	NAA+NAAG	5.64/0.75	5.44/0.72	6.06/1.09
Cho	1.38/0.16	1.34/0.22	1.43/0.20	Cho	1.58/0.18	1.54/0.17	1.71/0.25
Cr	5.06/0.60	4.80/0.38	5.06/0.60	Cr	4.96/0.54	4.70/0.64	5.25/0.86

HC: Healthy controls SCZ−: 22q11DS without psychosis SCZ+: 22q11DS with psychosis.

Glu: glutamate Gln: glutamine Glx: Glu+Gln NAA: *N*-acetylaspartate.

NAA+NAAG: NAA+*N*-acetylaspartylglutamate mI: myo-inositol Cr: creatine Cho: choline.

Metabolite concentrations are expressed in millimoles per liter.

a *P*<0.05 for SCZ− *vs.* SCZ+.

b *P* = 0.05 for HC *vs.* SCZ+.

### Plasma Proline and Plasma Glutamine

For the whole 22q11DS group, the mean±SD for plasma proline was n = 13, 354±128.88 µmol/l and for plasma glutamine n = 8, 540.62±68.14 µmol/l. The correlation between these variables was not significant (n = 8 ρ = 0.26 *P* = 0.53). The normal laboratory range for plasma proline was 77–343 µmol/l and for plasma glutamine 344–743 µmol/l.

There were no significant differences between 22q11DS SCZ− and 22q11DS SCZ+ for plasma proline (22q11DS SCZ− n = 8, 376.37±145.64 µmol/l, 22q11DS SCZ+ n = 5, 318.20±100.56 µmol/l; *P* = 0.56) or plasma glutamine (22q11DS SCZ− n = 4, 555.25±79.47 µmol/l, 22q11DS SCZ+ n = 5, 540.80±63.70 µmol/l; *P* = 0.78). There was no significant correlation between plasma proline and plasma glutamine in any of the two 22q11DS groups.

The correlation between DLPFC glutamate and plasma proline for the whole 22q11DS group was not significant (n = 11 ρ = 0.26 *P* = 0.43). Also, there was no significant correlation between proline and DLPFC glutamate for the 22q11DS SCZ− (n = 5 ρ = 0.30 *P* = 0.62) and 22q11DS SCZ+ group (n = 6 ρ = 0.37 *P* = 0.47).

The correlation between hippocampal glutamate and plasma proline for the whole 22q11DS group was not significant (n = 10 ρ = 0.21 *P* = 0.56). There was no significant correlation between proline and hippocampal glutamate for the 22q11DS SCZ− (n = 6 ρ = 0.03 *P* = 0.96) and 22q11DS SCZ+ group (n = 4 ρ = 0.40 *P* = 0.80).

## Discussion

In this first *in vivo* ^1^H-MRS study in 22q11DS we measured metabolite concentrations of the DLPFC and hippocampal region in adults with and without schizophrenia and in healthy controls. Our main findings are increased hippocampal glutamate and myo-inositol concentrations in 22q11DS SCZ+ compared to 22q11DS SCZ−. Metabolites of the DLPFC did not differ significantly across the groups.

^1^H-MRS studies of the hippocampus in schizophrenia have shown ambivalent results concerning glutamate; some studies reported no alterations of glutamate concentrations in subjects experiencing prodromal symptoms of schizophrenia [37] or in chronic schizophrenia [38], [39]. Other studies reported increased hippocampal glutamate in patients with schizophrenia [40] or a tendency towards increased glutamate in a group of medicated first episode patients [41].

In the present ^1^H-MRS study we found increased concentration of glutamate and Glx in the hippocampal region of 22q11DS SCZ+ compared to 22q11DS SCZ−. Also, hippocampal Glx was increased in 22q11DS SCZ+ compared to healthy controls. Excessive release of glutamate and consequent overstimulation of postsynaptic receptors might have an influence on the cognitive and psychotic symptoms associated with the NMDA hypofunction in schizophrenia [25]. In line with this observation and in agreement with previous research in schizophrenia, our finding of increased hippocampal glutamate in 22q11DS SCZ+ suggests that glutamate disturbance may be underlying psychotic symptoms in 22q11DS SCZ+. Moreover, the 22q11DS SCZ+ had overall lower IQ than 22q11DS SCZ−. Increased hippocampal glutamate could also explain the cognitive impairment in 22q11DS SCZ+ since this brain area is involved in learning and memory functions. Although speculative, increased hippocampal glutamate in 22q11DS SCZ+ might also indicate NMDA receptor hypofunction in this group.

Glutamate neurotransmission may in part be influenced by proline. Increased concentrations of proline associated with hyperprolinemia type II (proline levels 10–15 fold above normal) have been shown to potentiate glutamate transmission in hippocampus and cerebral cortex [15], [42]. Hyperprolinemia of the type I has been observed in patients with 22q11DS (plasma proline levels with a range of 3–10 fold above normal) which results from inherited deficiency of POX enzyme [12], [30]. In the present study half of the 22q11DS patients had elevated proline levels. Contrary to our expectation, we found similar proline levels in 22q11DS SCZ+ and 22q11DS SCZ−. Increased proline levels may depend on genetic variation of the PRODH allele [8] or on interaction with other genes. For instance, a study of hyperprolinemia in 22q11DS showed an association between hyperprolinemia and psychosis in 22q11DS patients only when Met, the low activity allele of the COMT gene, was taken into account [12]. We found no correlation between plasma proline, plasma glutamine and cerebral glutamate concentrations in the whole 22q11DS group or in 22q11DS SCZ− *vs.* 22q11DS SCZ+. Thus, although we found increased hippocampal glutamate concentrations in 22q11DS SCZ+, its underlying mechanisms remain unclear.

In addition to increased hippocampal glutamate, we found higher concentrations of myo-inositol in 22q11DS SCZ+ compared to 22q11DS SCZ−. Increased concentrations of myo-inositol have previously been reported in mild cognitive impairment and Alzheimer disease [43], [44]. Also in Down syndrome increased hippocampal myo-inositol has been associated with reduced cognitive ability [45]. Changes in myo-inositol levels may reflect abnormalities in membrane metabolism, in intracellular signaling mechanisms, neuronal development and survival [46]. Hence, increased myo-inositol may explain part of the hippocampal brain abnormalities and learning disabilities seen in 22q11DS SCZ+.

The finding of increased glutamate and myo-inositol may be tightly related to each other in the psychopathology in 22q11DS SCZ+. Myo-inositol is primarily found in astrocytes [47] which interact with neurons and play a critical role in the synthesis of glutamate [48], [49]. Elevated concentration of myo-inositol may indicate increased number or increased metabolic activity of astrocytes. Astrocyte dsysregulation in turn may trigger increased glutamate uptake and glutamate-glutamine cycling conversion. This could reflect altered glutamatergic neurotransmission in this genetic predisposed group, which combined with environmental interaction may increase the vulnerability for development of schizophrenia.

We found no significant variation in neurometabolites concentration between the whole 22q11DS patient group and the healthy control group. This might be explained by group differences in the proportion of gray matter/white matter within the DLPFC and hippocampal voxels. Also, we found no evidence for altered glutamate in the DLPFC of 22q11DS patients (22q11DS SCZ+ *vs.* 22q11DS SCZ−) *vs.* healthy controls. In patients with chronic schizophrenia, ^1^H-MRS studies of the frontal cortex have shown increased [40], [50], [51] and reduced glutamate concentrations [29], [39], [52], [53]. Perhaps, brain dysfunction associated with psychosis in 22q11DS involves specific regions of the temporal lobe [54], [55]. Furthermore, it is also possible that abnormalities in glutamatergic function in this brain region may exist at the level of NMDA receptor or in second messenger signaling without alterations in glutamate concentration.

An interesting observation is that most of the metabolite contents are in the order of 22q11DS SCZ−<HC<22q11DS SCZ+. We are not aware of an existing explanation for this relation in the literature. However, we hypothesize that prior to the development of schizophrenia patients with 22q11DS in general may have decreased neuronal metabolism as has been observed for glutamate in individuals with increased vulnerability to schizophrenia (at risk mental state - ARMS) [56], [57]. On the other hand, an instable neuronal metabolism may predispose a subgroup of 22q11DS patients to psychotic decompensation. Another possibility is that higher metabolites in the 22q11DS patients are the result of the transition to psychosis instead of the cause. This would mean that high metabolic rates in 22q11DS are state- instead of trait-related. Due to the cross-sectional design of our study we are unable to confirm this hypothesis. Longitudinal research in 22q11DS patients before and after transition to psychosis is therefore warranted.

The strengths of this study include the evaluation of neuronal integrity in 22q11DS according to psychiatric status of 22q11DS SCZ− and 22q11DS SCZ+ and in comparison to age matched healthy controls. Also, all MRS spectra were carefully inspected and were included only if fulfilling the quality criteria of LCmodel.

We have to acknowledge some limitations of the study; unfortunately at the time of the study we were not able to analyze plasma samples of proline and glutamine of healthy controls. Future studies with large sample sizes including healthy volunteers, should elucidate the relationship between plasma levels (of proline, glutamate, glutamine), cerebral metabolites and the vulnerability to schizophrenia. We did not determine the size of deleted region in each 22q11DS patient although the majority of patients have a typically deleted 3 Mb region. We did not to apply a correction for multiple comparisons because the possibility of inflating type II error [58]. Since increased hippocampal glutamate possibly corroborates the involvement of glutamate in psychosis [11], [41], [59] and converging evidence from animal and human studies propose the hippocampus as crucial brain area involved in the vulnerability to schizophrenia [60], [61] we chose to avoid a too stringent evaluation. We were not able to determine tissue contributions to measured metabolites; the use of unsegmented voxels (*i.e.*, assessment of metabolite concentrations without addressing the impact of different tissue included in the voxel of interest) may increase the standard error of measurement and diminish the power to detect significant differences. The cubic shape of hippocampal voxel may have allowed for contamination signals from adjacent regions of the hippocampus. Moreover, the effect of medication can be a potentially confounding factor in ^1^H-MRS studies [62]. In our study, antipsychotic drugs may have affected metabolites concentrations of frontal lobe in 22q11DS SCZ+. In fact, in the DLPFC, unlike in the hippocampus, we found a significant positive correlation between dosage of medication and glutamine concentration and a trend towards positive correlation between dosage of medication and Glx concentration in 22q11DS SCZ+ patients. This may also indicate that antipsychotics modulates neuronal metabolism in a regionally specific fashion.

Due to similar chemical components glutamate and glutamine overlap significantly in the ^1^H resonance spectrum. The use of higher field strengths and implemented spectroscopy analysis technique makes it possible to improve glutamate quantification. Discrepancies across earlier ^1^H-MRS studies that proposed to investigate glutamate in psychosis could have resulted from differences in brain regions of interest, patient population and stage of disease or issues of spectroscopy measurements.

In conclusion, our findings suggest vulnerability of the hippocampus in the psychopathology of 22q11DS SCZ+. Although the generalizability of the results is restricted by the relatively small sample size, altered glutamate and myo-inositol metabolism may partially explain the psychotic symptoms and cognitive impairments seen in this group of patients. Future ^1^H-MRS studies with larger sample sizes including other prefrontal and temporal brain regions will help to clarify brain metabolism and integrity in 22q11DS.
