# Correction: Proton Magnetic Resonance Spectroscopy in 22q11 Deletion Syndrome

**DOI:** 10.1371/annotation/805ba3f5-0fd7-41f9-bc2c-31ebf507f05b

**Published:** 2011-08-22

**Authors:** Fabiana da Silva Alves, Erik Boot, Nicole Schmitz, Aart Nederveen, Jacob Vorstman, Christina Lavini, Petra Pouwels, Lieuwe de Haan, Don Linszen, Therese van Amelsvoort

The correct spelling of the 7th author's name is: Petra J. Pouwels. 

